# Acute and chronic effects of Titanium dioxide (TiO_2_) PM_1_ on honey bee gut microbiota under laboratory conditions

**DOI:** 10.1038/s41598-021-85153-1

**Published:** 2021-03-15

**Authors:** G. Papa, G. Di Prisco, G. Spini, E. Puglisi, I. Negri

**Affiliations:** 1 Department of Sustainable Crop Production-DIPROVES, Università Cattolica del Sacro Cuore, Via Emilia Parmense 84, 29122 Piacenza, Italy; 2 Institute for Sustainable Plant Protection, National Research Council, Piazzale Enrico Fermi 1, 80055 Portici, Naples, Italy; 3 CREA Research Centre for Agriculture and Environment, Via di Corticella 133, 40128 Bologna, Italy; 4 Department of Agricultural Sciences, University of Napoli Federico II, Via Università 100, 80055 Portici, Italy; 5 Department for Sustainable Food Process-DISTAS, Università Cattolica del Sacro Cuore, Via Emilia Parmense 84, 29122 Piacenza, Italy

**Keywords:** Entomology, Microbial ecology, Environmental impact

## Abstract

*Apis mellifera* is an important provider of ecosystem services, and during flight and foraging behaviour is exposed to environmental pollutants including airborne particulate matter (PM). While exposure to insecticides, antibiotics, and herbicides may compromise bee health through alterations of the gut microbial community, no data are available on the impacts of PM on the bee microbiota. Here we tested the effects of ultrapure Titanium dioxide (TiO_2_) submicrometric PM (i.e., PM_1_, less than 1 µm in diameter) on the gut microbiota of adult bees. TiO_2_ PM_1_ is widely used as a filler and whitening agent in a range of manufactured objects, and ultrapure TiO_2_ PM_1_ is also a common food additive, even if it has been classified by the International Agency for Research on Cancer (IARC) as a possible human carcinogen in Group 2B. Due to its ubiquitous use, honey bees may be severely exposed to TiO_2_ ingestion through contaminated honey and pollen. Here, we demonstrated that acute and chronic oral administration of ultrapure TiO_2_ PM_1_ to adult bees alters the bee microbial community; therefore, airborne PM may represent a further risk factor for the honey bee health, promoting sublethal effects against the gut microbiota.

## Introduction

Honey bees (*Apis mellifera* Linnaeus) are important providers of ecosystem services, both regulating, through pollination of a wide range of crops and uncultivated plants, and provisioning, for the delivery of honey, pollen, propolis and other products to humans. Moreover, the honey bee is an important bioindicator of environmental contamination, and both the insect and its products are used for the detection of environmental pollutants^1–5^.

In the last decade, one of the major problems plaguing honey bees is a phenomenon named Colony Collapse Disorder (CCD) which causes loss of colonies worldwide^6–8^. The multifactorial origin of CCD is widely acknowledged, and environmental stressors such as pesticides, heavy metals, or airborne particulate matter (PM) pollutants may play a key role in driving the bees’ decline^9^.

While the effects of pesticides and heavy metals on bees are widely recognised, till now very few data are available on the effects of PM on bees’ health. These studies include lethal and sublethal effects (e.g., behaviour, gene expression, and cellular alterations) of the exposure to lead and cadmium oxides and TiO_2_ nanoparticles^10–13^.

In polluted environments, airborne PM is known to stuck to the body of the bee and can be also ingested through contaminated pollen and honey that represent the food sources of the bee colony^3,5,14^. If ingested, dusts can come into contact with the gut microbiome lining the intestinal epithelium posing a hazard to the bacterial community. Recent evidence suggests that environmental stressors can indirectly compromise bee health through gut microbiota disruption^15^. The honey bee harbors a simple and distinct gut community, thought to be the result of a long-lasting evolutionary relationship^15^. While no evidence has been reported until now on the impacts of PM on the bee health, alterations of the gut microbial community composition were demonstrated in bees exposed to antibiotics^16^, glyphosate^17^, insecticides^18,19^ and sublethal doses of cadmium and selenite^20^.

The gut of worker bees is dominated by nine clusters of bacterial species comprising between 95% and 99.9% of total diversity in almost all individuals, based on investigations carried out on 16S rDNA^21–23^ and on total DNA metagenomics of intestinal samples^24^. The two omnipresent Gram-negative species are *Snodgrassella alvi* and *Gilliamella apicola*, both members of Phylum Proteobacteria^25^. Among Gram-positive bacteria, two groups of species in the Firmicutes phylum are ubiquitous and abundant, referred to as the *Lactobacillus* Firm-4 and *Lactobacillus* Firm-5 clades^26^. Although often less abundant, the cluster of the species *Bifidobacterium asteroides*^27^ is also found in most adult worker bees. Less numerous and even less prevalent are the Proteobacteria species *Frischella perrara*^28^, *Bartonella apis*^29^, *Parasaccharibacter apium*^23^ and a group of Gluconobacteria species designated Alpha2.1. These species have narrow niches in the intestines of bees (e.g., *F. perrara*) or are generalists, i.e., they are also found in the hive environment (for example, *P. apium*, *Lactobacillus kunkeei* and the Alpha2.1 group), which may explain their relatively lower frequency in bee gut detections. While other bacteria may occasionally be present, these nine species groups represent bacterial lineages that appear to be specifically adapted to life alongside their hosts, bees. This gut microbiota organization is well described by Kwong & Moran^30^.

The newly hatched larvae are free of bacteria that start colonizing the gut thanks to interactions with worker bees and the hive environment^31,32^. During the metamorphosis of the larvae to pupae and finally, into adult bees, the lining of the intestine is renewed: the newly emerged adult bees have very few bacteria in the intestine and are readily colonized by the typical intestinal microbial community^33^.

Titanium dioxide (TiO_2_) is a naturally occurring metal oxide. As rutile, TiO_2_ is not rare in nature and may concentrate in the heavy fraction of sediments. Synthetic TiO_2_ in form of sub-micrometric PM, i.e., PM_1_, less than 1 µm in diameter, is widely used as a filler and whitening agent in a range of manufactured objects, such as plastics, paints, paper, printing inks, textiles, catalysts, floor and roofing materials, and vehicles components. TiO_2_ is also a common ingredient in cosmetics, pharmaceuticals, sunscreen and as a food additive. While TiO_2_ used for non-food applications has surface coatings of alumina and silica, to reduce photoactivity, and organic surface treatments, conferring hydrophobic properties, TiO_2_ as a food additive is ultrapure, and PM size ranges from 400 µm to 30 nm^34,35^.

The potential ecological and human health impact of exposure to TiO_2_ is of growing concern. Indeed, inhalation and intra-tracheally administration of PM of TiO_2_, including nano-meter sized particles, induces lung cancer in rats^36^. TiO_2_ has indeed been classified by the International Agency for Research on Cancer (IARC) as a possible human carcinogen in Group 2B (IARC, 2010). New evidence suggests that oral exposure to nanosized TiO_2_ promotes chronic intestinal inflammation and carcinogenesis in rats^35^, as well as dysbiosis of gut microbiota^37^.

Trace element analyses already demonstrated that Titanium can be detected in honey, pollen and bees, especially in high density urban and residential/suburban areas^38–41^. Titanium can be only found in combined form and the most widespread chemical form both from natural and anthropogenic sources is TiO_2_. Therefore, honey bees may be severely exposed to TiO_2_ ingestion through contaminated honey and pollen, and in severely polluted areas, PM_1_ of TiO_2_ can be detected in pollen grains and honey (Negri, personal communication).

The toxicity of TiO_2_ PM_1_at nano-scale has been already studied in honey bee with histological and immunohistochemical approaches^12^ shedding light on the negative effects at high doses. However, in-depth studies on the effects on the gut environment of the TiO_2_ ingested by bees are still lacking, and no studies on specific impacts on the bee’s gut microbiota are available. Here we wanted to fill this gap by studying the effect of TiO_2_ on honey bees gut microbiota in the experimental context of controlled oral administration, providing evidence that ingestion of PM_1_ of TiO_2_ can alter the bee microbial community. Sub-lethal doses were employed, while acute and chronic exposures were assessed at 24 and 96 h post-emergency for acute and chronic experiments respectively.

## Results

### SEM–EDX results

Scanning electron microscope (SEM) coupled with X-ray (EDX) analyses were used to assess the morphology, chemical composition and size of TiO_2_ particles delivered to the bees.

**Figure 1:**
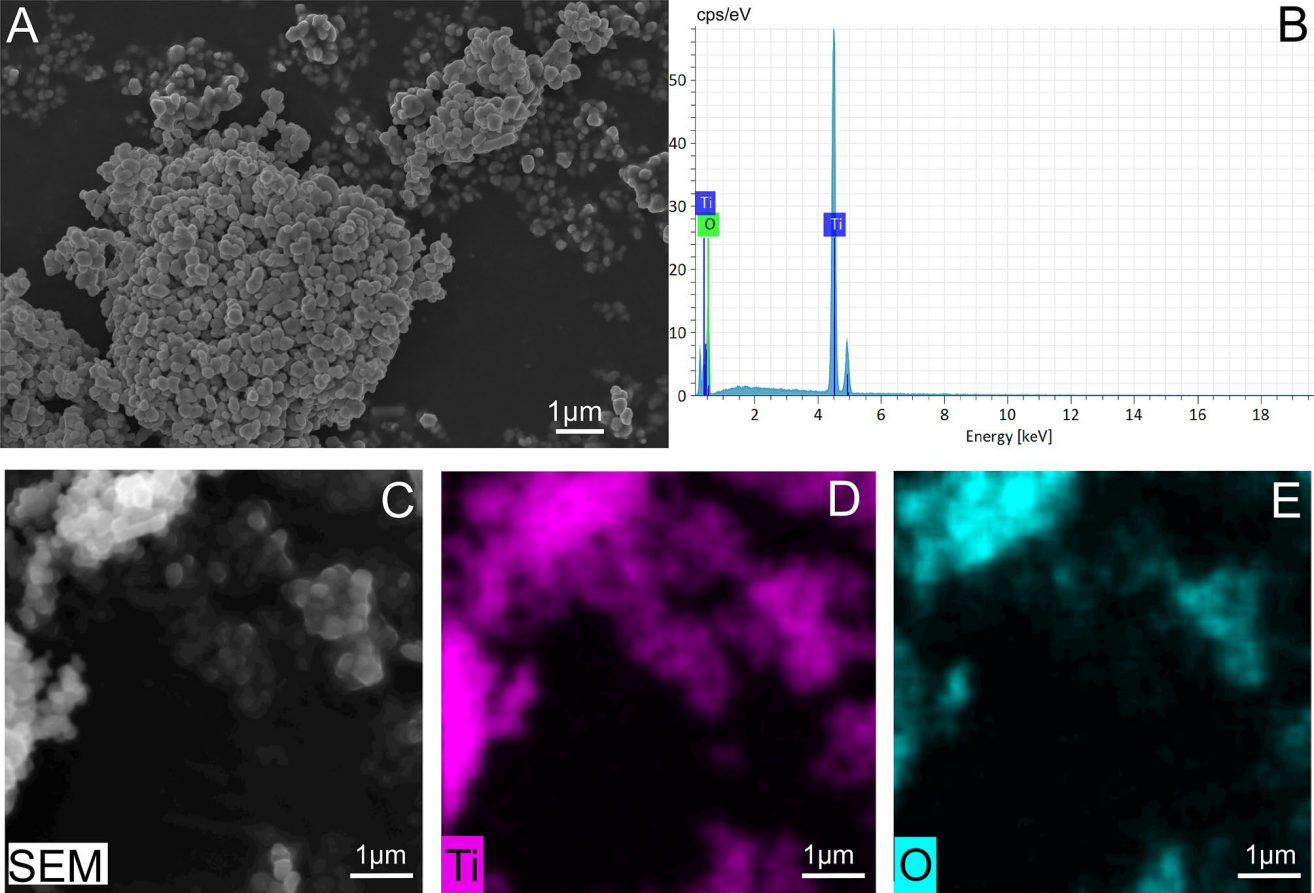
Control sample of TiO_2_ rutile powder and elemental mapping. (**A**) SEM image showing the morphology and size and (**B**) EDX spectrum showing the purity of the TiO_2_ powder. (**C**) SEM-BSE image and the (**D**) titanium and (**E**) oxygen element maps.

SEM analyses showed that the TiO_2_ stock powder had a sub-micrometre size < 1 µm (between 800 and 200 nm) and an ellipsoidal/spherical shape (Fig. 1A). Moreover, EDX analyses and compositional mapping confirm the purity of TiO_2_ rutile (Fig. 1B–E).

SEM–EDX analyses demonstrated the absence of TiO_2_ in haemolymph collected from the acute, chronic and control samples (Figure S1A,B). In the haemolymph, EDX spectra (Figure S1B) showed the presence of many elements e.g., Mg, Ca, Na, S, P, K, Cl^42^.

**Figure 2:**
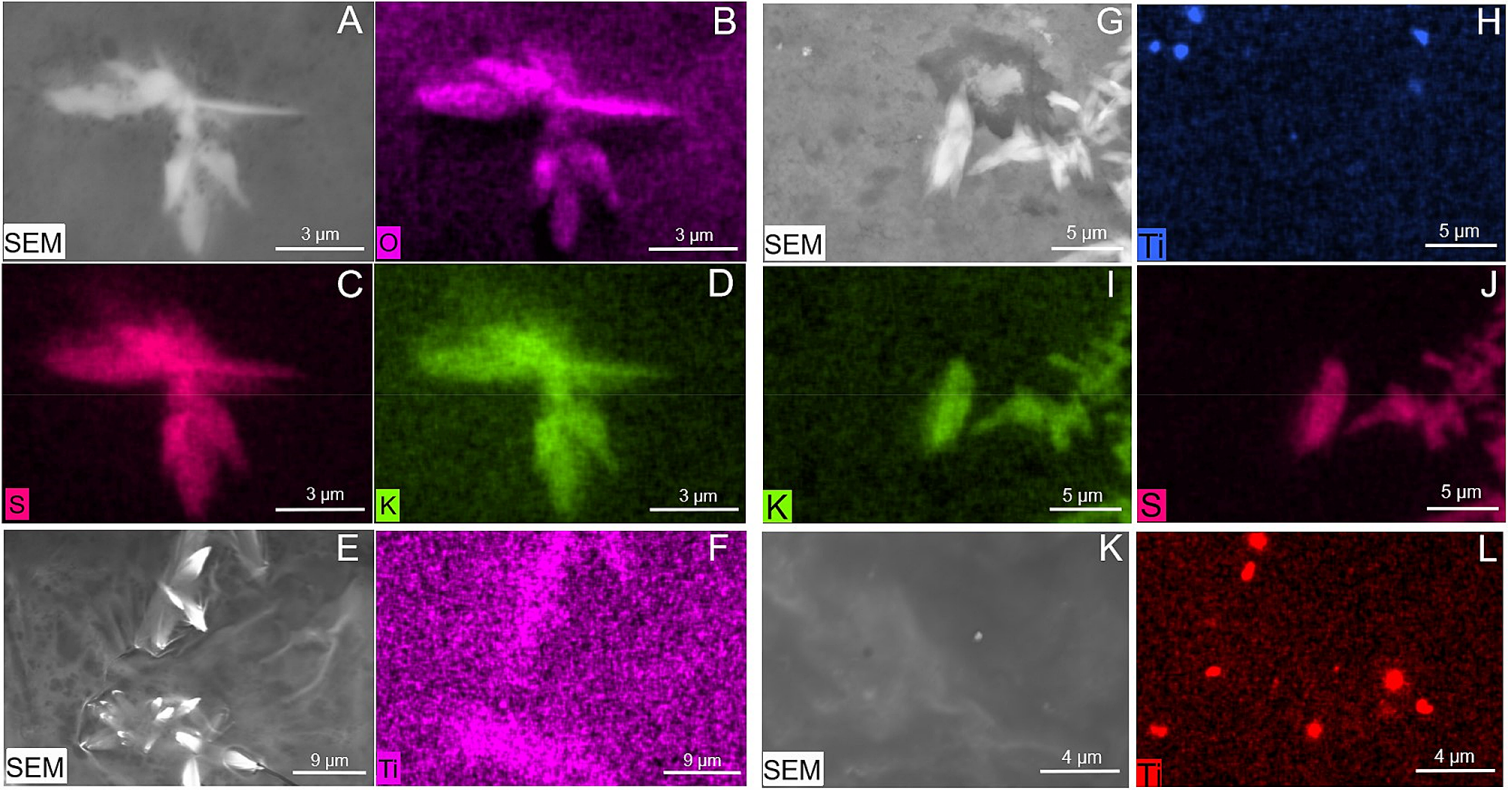
Elemental mapping of the rectum in control (**A**–**D**) and chronical samples (**E**–**L**). (**A**,**E**,**G**,**K**) SEM-BSE images; (**B**–**D**) element analysis highlighting the presence of K_2_SO_4_ crystals in the control sample; (**F**) titanium dioxide associated with K_2_SO_4_ crystals; (**H**–**L**) titanium dioxide not associated with K_2_SO_4_ crystals.

SEM observation performed on chronic and control gut highlighted the presence of many lanceolate crystals likely due to precipitation of salts. EDX analysis confirmed their chemical composition as K_2_SO_4_ (Fig. 2A–D). In the rectum of treated bees, TiO_2_ was found both associated (Fig. 2E,F) and not associated (Fig. 2G–L) with K_2_SO_4_ crystals.

### Bacterial diversity in the studied honey bees

The gut bacterial diversity was assessed by means of Illumina HTS of bacterial 16S amplicons covering the V3-V4 regions. A total of 537,641 sequences were produced, filtered and downscaled to 216,000 (i.e., 12,000 per each sample) after elimination of homopolymers, sequences not aligning to the target region, chimaeras, non-bacterial sequences and rarefaction to the least populated sample. After this downscaling, one out of 18 samples (a replicate of the 100X acute test) was eliminated because it had a lower number of sequences. The resulting Good’s index of coverage of the rarefied samples was 94.7 ± 1.2%, indicating that the vast majority of bees gut bacterial diversity was covered by the sequencing.

**Figure 3:**
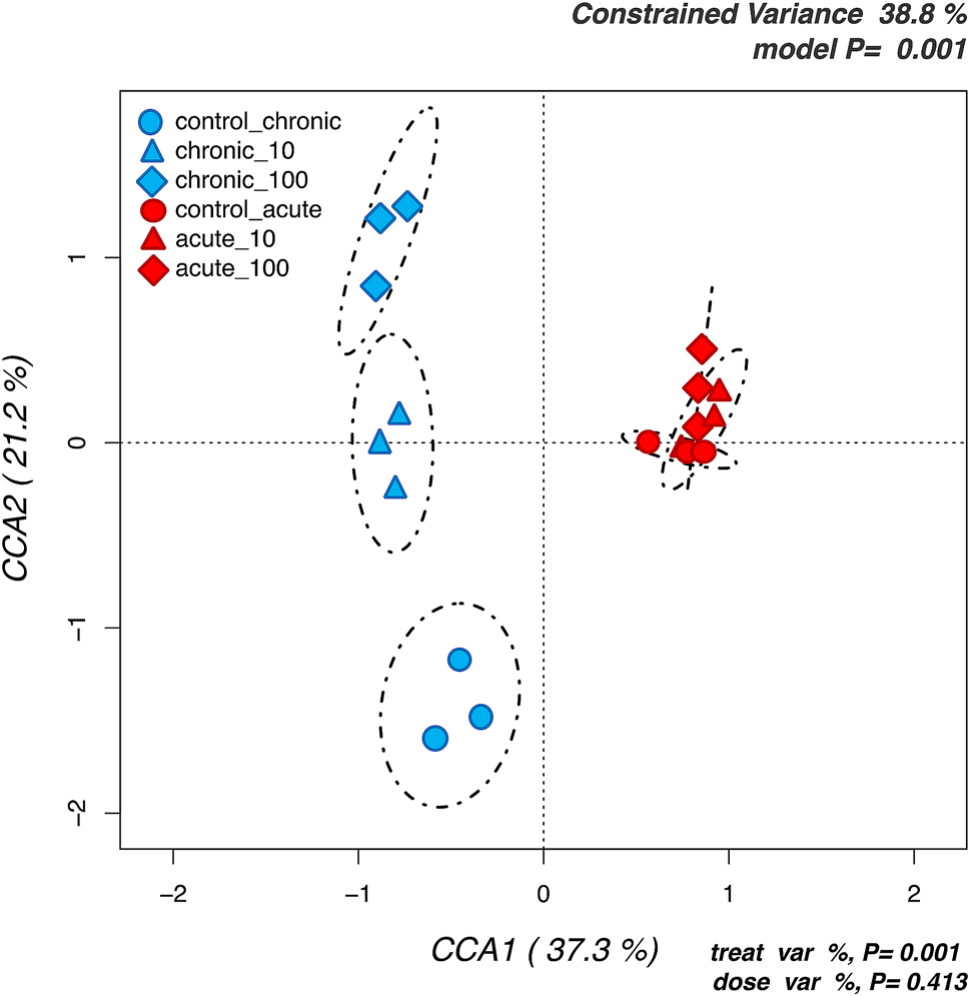
Hypothesis-driven canonical correspondence analysis (CCA) of honey bees gut microbiota testing the significance of the treatment and dose effects on the abundances of all analysed OTUs. Samples are labelled according to the 6 groups studied.

The structure of honey bee gut bacterial community was investigated at OTUs level, testing with a CCA model if the treatment (control vs chronic vs acute) and the dose had significant effects on the bacterial gut communities of the studied bees. Results (Fig. 3) indicated that the bees from the acute and the chronic experiments, being sampled at different life stages, hosted very different gut microbiota. Within each exposure time, it was found that samples were clearly grouped among doses for chronic exposure, but not for the acute. Similar outcomes were obtained by Principal Component Analyses (Figure S2).

**Figure 4:**
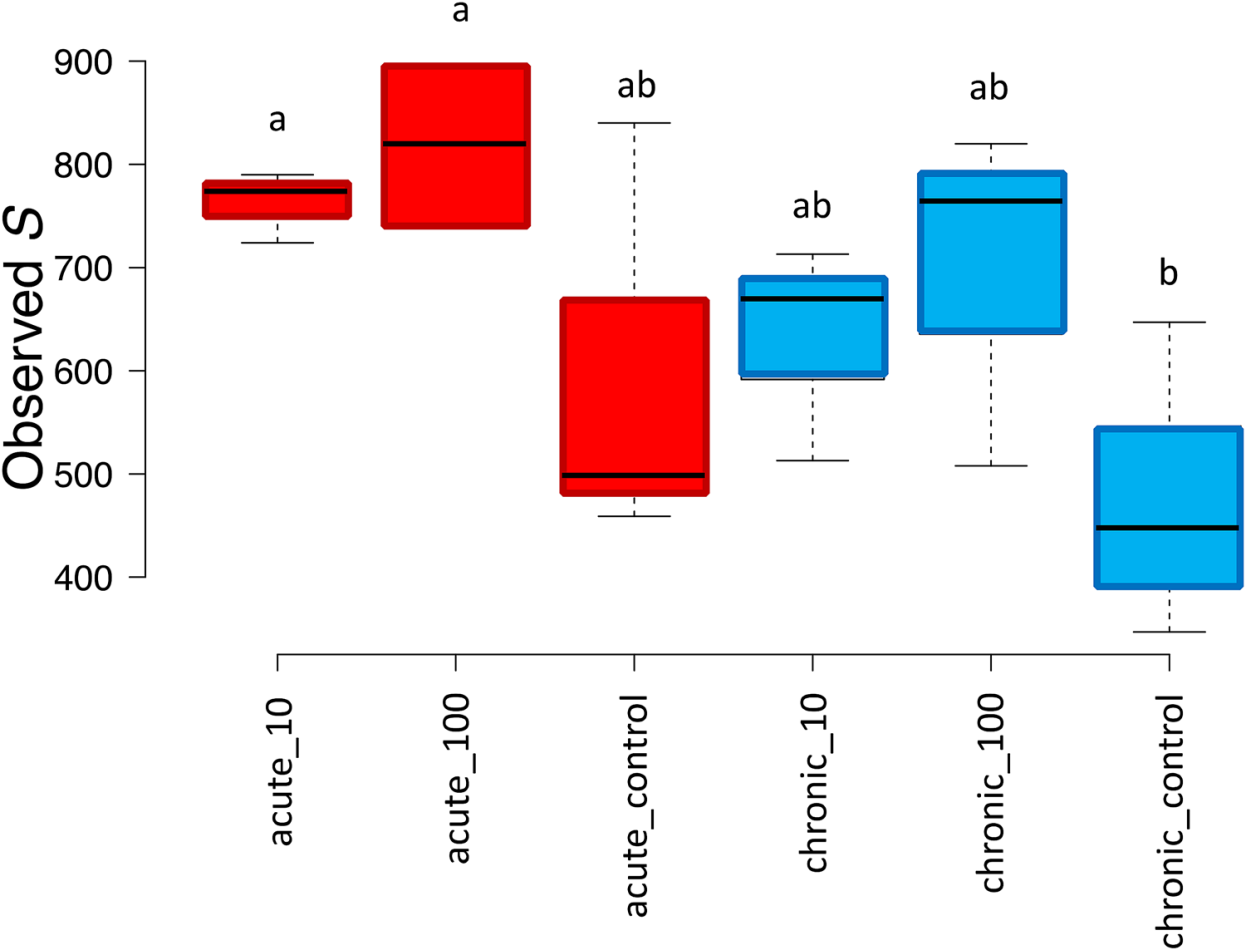
Number of observed OTUs (S) for the chronic and acute treatments and their respective controls. Significant differences are highlighted with different letters according to Tukey’s HSD test for comparison of means.

Differences among samples were also reflected by α-diversity analyses on the total number of OTUs identified in each treatment, which highlighted a clear trend: bees exposed to both chronic and acute TiO_2_ PM displayed a higher diversity as compared to their relative controls. The difference was significant according to LSD test between the chronic control and the two acute treatments (Fig. 4).

**Figure 5:**
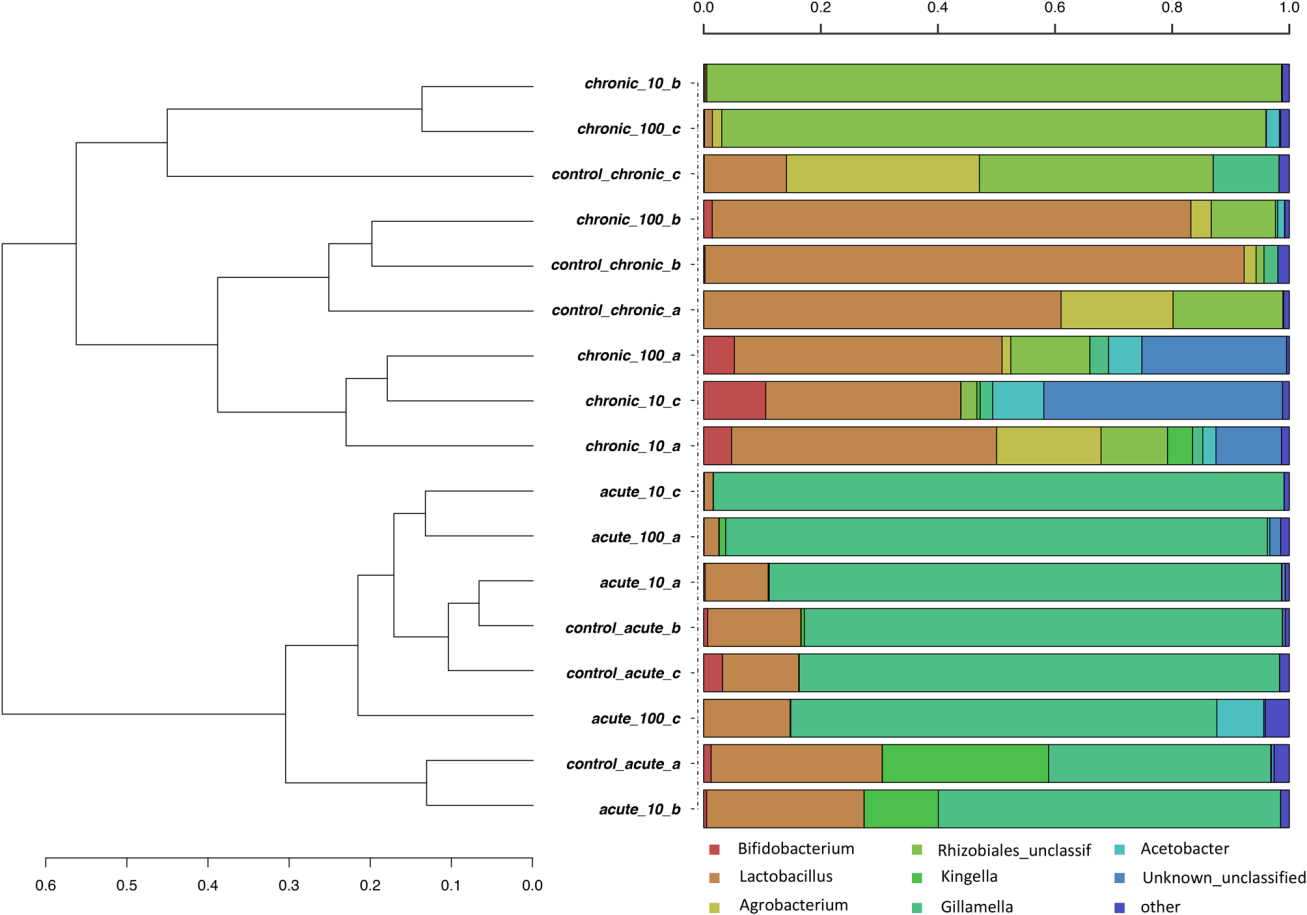
Hierarchical clustering of sequences classified at the genus level for both chronic and acute treatments. Bars of different colours indicated the relative percentage of the genera identified in the honey bees gut. Only genera participating with > 1% in at least one sample are shown, while taxa with lower participations were added to the “other” group. Similar samples were clustered using the average linkage algorithm.

The distinct bacterial community structures induced by TiO_2_ PM exposure were also highlighted by hierarchical clustering of sequences classified at the genus level (Fig. 5): in agreement with the CCA results, chronic and acute experiments were grouped in two main separate clusters sharing less than 70% of similarity. The two controls for each treatment representing adults sampled at two different life stages were also grouped separately one from the other. *Gilliamella* was the dominant genus in the acute experiments (24 h), with relative percentages reaching more than 80% of total abundance in a number of acute treatments at both 10X and 100X, followed by *Lactobacillus* and *Acetobacter*. A very different composition was identified in 96 h bees of the chronic exposure experiment (Fig. 5): here the dominant genus was *Lactobacillus*, which decreased in the TiO_2_ exposed bees. The latter was however enriched in *Bifidobacterium*, reaching relative concentration between 5 and 12% in a number of chronically exposed bees. A particular feature was detected in two treated replicates at 10X and 100X, with *Acetobacter* covering the vast majority (i.e., > 98%) of the observed diversity at the genus level.

### Distinctive features in the gut microbiota of honey-bees acutely exposed to TiO_2_

After defining that the two bee groups from the acute and the chronic exposure experiments had very different gut bacterial compositions (Figs. 3 and 5), separate analyses were carried out on the two groups, focusing on the relative presence of the most abundant OTUs classified at the species level.

**Figure 6:**
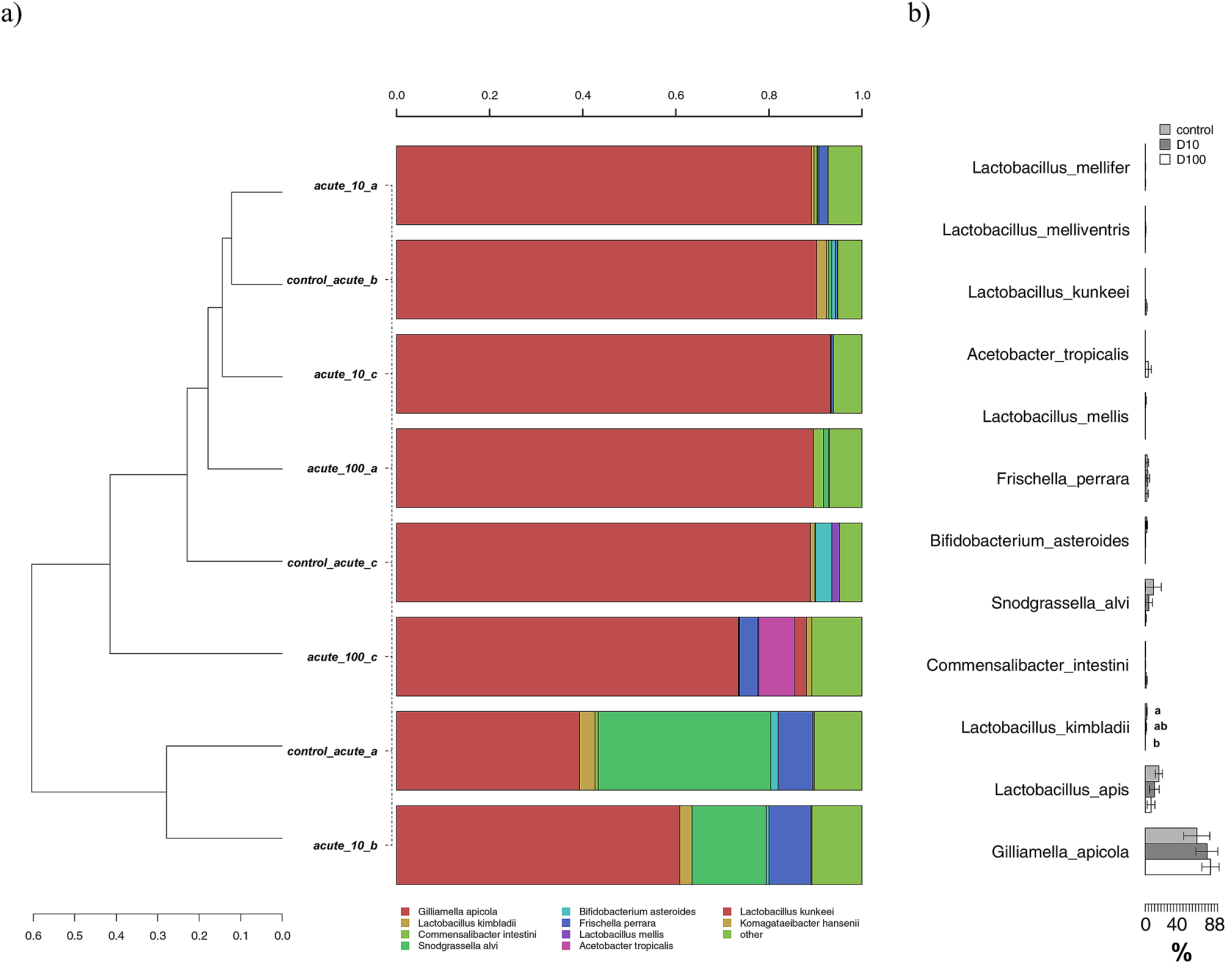
(**a**) Hierarchical clustering of sequences of OTUs participating with > 1% in at least one sample for the acute exposure experiment. Rare OTUs with lower participation were added to the “other” group; (**b**) metastats model showing differences among treatments for the most abundant OTUs. Significant differences are highlighted with different letters according to Tukey’s HSD test for comparison of means.

In the acute exposure experiments, *G.apicola* was the most abundant species with an average of ca 70% of the total bacterial community, followed by *Lactobacillus apis*, *S. alvi*, *Lactobacillus kimbladii* and *Acetobacter tropicalis* (Fig. 6a). The clustering did not show clear discrimination between control and treated bees, but a Metastats model on the same OTUs revealed a number of significant differences (Fig. 6b). Specifically, a significant reduction with increasing acute doses of TiO_2_ was found for *L. kimbladii*. The same trend was detected for *L. apis* and *S. alvi*, but with no statistical significance.

### Distinctive features in the gut microbiota of honey-bees chronically exposed to TiO_2_

**Figure 7:**
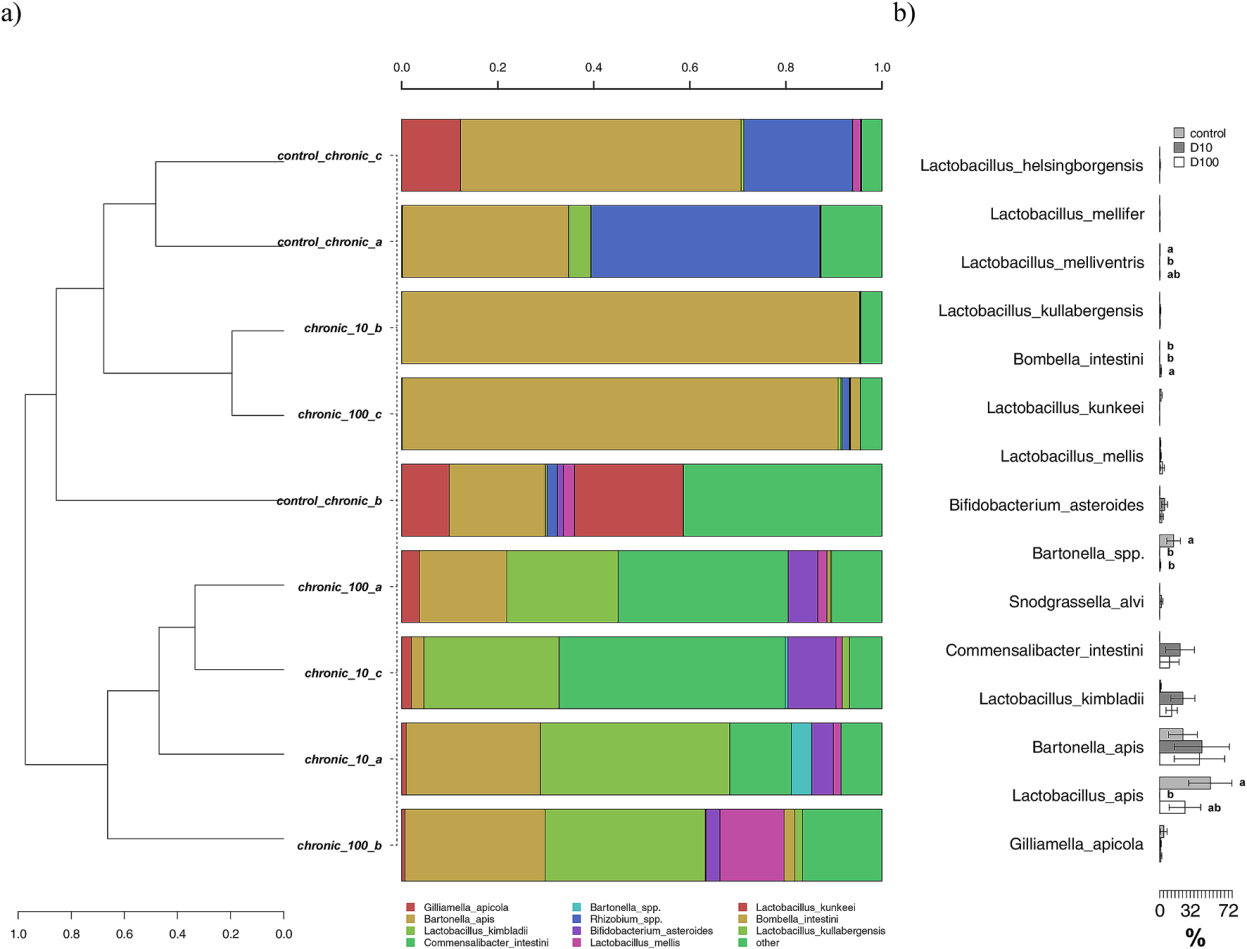
(**a**) Hierarchical clustering of sequences of OTUs participating with > 1% in at least one sample for the chronic exposure experiment. Rare OTUs with lower participation were added to the “other” group; (**b**) metastats model showing differences among treatments for the most abundant OTUs. Significant differences are highlighted with different letters according to Tukey’s HSD test for comparison of means.

A stronger differentiation between control and TiO_2_ exposed bees was found in the chronic experiments. Here, the composition at the species level was very different, reflecting the age differences between this and the previous group of bees. *G. apicola* was still present, but with much lower relative abundances (< 2%). On the contrary, a strong increase in abundances was found for *B. apis*, *L. apis*, *L. kimbladii*, *Commensalibacter intestini*, and *Bartonella* spp. (Fig. 7a). Much higher was also the number of species displaying significant differences between treatments and controls (Fig. 7b), thus highlighting significant effects of chronic TiO_2_ exposure on the honey bees gut microbiota. Specifically, *L. apis*, *Lactobacillus melliventris* and *Bartonella* spp. were significantly reduced by the exposure to TiO_2_, in most cases with a dose dependent effect. On the contrary, *Bombella intestini* was found to be significantly enriched only in the X100 treatment.

## Discussion

In the present study, we explored the potential effects of acute and chronic oral administration of pure TiO_2_ rutile on honey bee gut microbiota. The particles used were PM_1_; ranging from 800 to 200 nm as characterized by SEM–EDX analysis. In a preliminary assay, we fed newly emerged worker bees with four different concentrations of TiO_2_ in 1 M sucrose solution, including 100 ng/µL and 10 ng/µL as test doses, after the rejection of the two higher doses 10^4^ ng/µL and 10^3^ ng/µL. Newly emerged worker bees were orally exposed to the selected doses of TiO_2_ in an acute (single treatment, sampling at 24 h) and a chronic application (treatments repeated every 24 h, sampling at 96 h). In both assays, we obtained no mortality for the entire trials. This is partly in contrast with similar experiments on the honey bee, that however applied higher doses than the one tested here: TiO_2_ resulted highly toxic at 1000 ng/µL^12^, while in another study the LC50 at 96 h resulted in 5.9 ng/µL^13^. Nevertheless, TiO_2_ showed high mortality at a very high dose of 2400 ng/µL) in cutworm (*Spodoptera litura*)^43^.

Unfortunately, the studies by Özkan et al.^13^ and Ferrara et al.^12^ do not provide a specific characterization of the size, morphology and purity of TiO_2_ dust delivered to the bees. In our research, TiO_2_ dust were carefully characterised by SEM/EDX, demonstrating the absence of contaminants or coatings and the dimensions greater than 200 nm. Such size might also explain the absence of TiO_2_ PM in the haemolymph since particles might not be able to cross the barrier of the gut epithelium. However, a more in-depth study involving the analysis of the gut epithelium should be carried out to exclude the presence of TiO_2_ PM from the epithelial cells or cytological abnormalities.

An indirect impact of TiO_2_ on haemolymph has been thoroughly investigated in other organisms. In *Mytilus galloprovincialis*, TiO_2_ has been shown to affect several immune parameters in both circulating haemocytes and haemolymph serum, resulting in immunomodulation^44–46^. In larvae of *Galleria mellonella* (Lepidoptera: Pyralidae), exposure with dietary TiO_2_ nanoparticles (NPs) has dose-dependent toxic effects and can enhance the stress-resistant capacity with a significant increase in the total protein amount and content of malondialdehyde (MDA) and glutathione S-transferase activity at 100, 500 and 1000 ppm^47^. In our experiments, SEM–EDX reveals the typical haemolymph elements spectrum (Mg, Ca, Na, S, P, K, Cl) of the honey bee^48^.

The analyses of bacterial community composition allowed by HTS of 16S amplicons clearly show that the acute and chronic exposed bees populations had hosted very different bacterial gut populations (Figs. 5, 6 and 7). This is expected since analyses were carried out after 1 dpe (day post-emergence) for the acute experiment and at 4 dpe for the chronic experiment. The β-diversity analyses here carried out by means of unconstrained (PCA and hierarchical clustering, Fig. 5 and Figure S2) and constrained (CCA, Fig. 3) analyses showed a clear distinction in bacterial community structure between the chronic and the acute experiments, and in the chronic experiments among controls and treated bees. These results are in line with several studies on other chemical stressors^16–20^, thus confirming that also PM modulates the honey bees gut bacterial community.

The results obtained in the unexposed controls are quite in agreement with previous evidence and showed the typical community of adult worker bee microbiota that is dominated by *Lactobacillus*, *Bifidobacterium*, *Gilliamella*, *Snodgrassella*. The presence of *Bartonella* and *Commensalibacter* in 4 dpe bees from the chronic experiment is in agreement with the characteristic gut microbial community of the so-called “winter bees”, i.e., the last generations of workers characterised by overwintering individuals with an extended lifespan to ensure colony survival until spring^49^. The variability occurring in control bees is in agreement with other studies, where it has been demonstrated that, while many of the phylotypes are consistently present in adult worker bees, their relative abundance can vary across individuals^49,50^, and representative of these two genera can be found also in early stages of development^20,51^.

In the treated populations, the two tested doses of TiO_2_ had significant impacts on the gut bacterial communities, with more differences between exposed and control bees in the chronic as compared to the acute exposure. As far as we know, this is the first study where the effects of TiO_2_ particles where specifically studied on bees gut microbiota, but they can be compared with a number of studies that previously assessed the detrimental impacts of airborne PM on the bees physiology^12,13^. Regarding α-diversity indexes, we found a dose-dependent increase in diversity, (Fig. 3), while the opposite was found for the neonicotinoid insecticide Thiacloprid^18^, polystyrene particles at µm levels^52^, and antibiotics^16^. It must be highlighted that in these cited works a significant mortality was detected, whereas in our study sub-lethal doses were tested and no mortality registered. It can thus be speculated that the microbiota responded to the sub-lethal stressors by increasing the diversity, as postulated by the ecological theory of the intermediate disturbance hypothesis firstly proposed by Connell in 1978^53^.

Concerning the changes in species abundances, we found that in the acute test only one species, *L. kimbladii*, was significantly reduced by the highest dose of TiO_2_ applied (Fig. 6). Firstly described in 2014 by Olofsson^54^ and colleagues, this species was more recently proposed as a possible probiotic^55,56^. Interestingly, *L. kimbladii* was instead found at higher abundances in the 96 h bees from the chronic exposure, with values higher than the control in the two treated theses (Fig. 7). This change may point to an adaptation in time of the microbial community, with a probiotic species becoming more abundant to counteract the chronic effects of a chemical stressor, as previously shown for *Bifidobacterium* species in bees exposed to the insecticide Nitenpyram^19^.

While in the acute experiment only *L. kimbladii* was significantly inhibited by the TiO_2_ particles (Fig. 6), in the chronic exposure three species were inhibited: *L. apis*, *L. melliventris* and *Bartonella* spp. (Fig. 7). *L. apis* is one of the most studied components of the bees gut microbiome, whose functions as probiotics were demonstrated by processes such as the attenuation of immune dysregulation^57^ and the inhibition of *Paenibacillus larvae* and other pathogens^58^; the induction of resistance towards bacterial infection was also demonstrated for *L. melliventris*, which was accordingly proposed as a bee probiotic^59^. Finally, the reduction of species belonging to *Bartonella* was also found in bees parasitized by *Varroa*,^60^, but this genus was also found to be increased after exposure to glyphosate^17^. The only taxon whose relative presence was significantly increased by chronic TiO_2_ exposure was *B. intestinii*, an acetic acid bacterium (AAB) firstly isolated in honey bees in 2017 by Yun and colleagues^61^. This species, together with other AAB, may play a role in the regulation of the innate immune system homeostasis^62^ and may thus represent an adaptation of the gut bacterial community to counteract the stressors sub-lethal effects.

Titanium oxide nanoparticles are known to exert a toxic effect on several bacteria, with proposed modes of action related to both chemical and physical interactions with the cells envelopes^63^, and alterations in human gut bacterial communities are often found including detrimental effects, as recently reviewed by Lams et al.,. (2020)^64^. Here we provide for the first time modulation of the honey bees gut microbiota induced by both acute and chronic exposure to TiO_2_ PM_1_, with stronger effects in the latter case. The effects we report are related to doses that are probably higher than the ones that can be found under field conditions and can be considered sub-lethal since no mortality was observed among all treated bees, and an increase in bacterial diversity was even found. On the other side, some negative effects related to the decrease in the relative percentages of some beneficial lactobacilli were however observed, and for this reason the role of airborne particulate as a further risk factor for *Apis mellifera* health in addition to other chemical stressors should be further explored.

## Materials and methods

### Honey bees

Experiments were conducted during October 2018 with *Apis mellifera ligustica* colonies maintained in the experimental apiary of the University of Napoli “Federico II”, Department of Agricultural Sciences. Brood frames with capped cells from two colonies were selected and kept in a climatic chamber at 36 °C and 60% relative humidity for approximately 16 h. Newly emerged workers were randomly selected and used for the bioassays.

### Preparation of TiO_2_ feeding solution

Titanium dioxide rutile (TiO_2_) powder was acquired from 2B Minerals S.r.l. (Modena, Italy). Suspensions were prepared by dissolving 0.5 g TiO_2_ in 50 mL distilled H_2_O, vortexed for ~ 20 s and sonicated for 15 min to increase dispersion and ensure the maximum distribution of particles in water^13^. A 1 M sucrose solution was then prepared with TiO_2_ suspensions.

Preliminary palatability tests on bees were carried out with the following dilutions: 10^4^ ng/µL, 10^3^ ng/µL, 100 ng/µL, 10 ng/µL. The last two concentrations were chosen for the experiments following rejection of the more concentrated solutions by the bees.

### Chronic and acute exposure

Two groups of 30 newly eclosed bees were randomly collected from the combs. The bees were fed ad libitum with 1 µL of 10 ng/µL and 100 ng/µL TiO_2_ solutions (1 M sucrose), respectively (hereinafter CH10- and CH100-bees), and then placed into plastic cages containing 1.5 mL of the same solution to which they were previously fed. Control bees consisted of 30 individuals fed ad libitum with an untreated sucrose solution (1 M).

The solution was changed every 24 h. During the experiments, all caged bees were kept in a climatic chamber at 36 °C and 60% relative humidity. After 96 h the bees were anesthetized with CO_2_ for ~ 30 s and the whole gut dissected as previously described^65^. Guts were stored in absolute ethanol and immediately refrigerated at -80 °C for the subsequent microbiological analysis. Experiments were conducted in triplicate (i.e., 3 replicates each made by three guts pooled together). Bees survival was recorded each day.

The experimental design of acute exposure was the same as in chronic exposure except for the duration of the experiment that in acute exposure lasted only 24 h instead of 96, and bees were fed only once with 10 ng/µL or 100 ng/µL TiO_2_ solutions (hereinafter AC10- and AC100-bees).

### TiO_2_ detection in the gut

To assess morphology, average size and chemical purity of TiO_2_ powder, few µgrams were mounted onto stubs and analysed through a Scanning Electron Microscope (SEM) provided with X-ray spectroscopy (EDX) (Zeiss Gemini SEM 500—Bruker Quanta X-Flash 61|31). Secondary Electrons (SE), BackScattered Electrons (BSE) images, and EDX point analyses, were acquired as previously described^3,14^.

The presence of TiO_2_ in the haemolymph and gut of chronic bees was investigated by SEM–EDX. Haemolymph from 4 randomly selected CH10- and CH100-bees plus 4 randomly selected control bees was sampled from the dorsal aorta, following the method standardized by Garrido and colleagues^66^. The bee gut (rectum with excrements) from CH10- and CH100-bees plus control bees (n = 4 randomly selected, for each concentration and control) were mounted onto stub and dried in a sterilized oven at 20 °C for 40 min. Samples were carbon coated and analyzed with SEM/EDX.

### Microbiota analysis

#### DNA extraction

DNA was extracted from gut samples collected for microbiological analyses. For each dose—acute and chronical—n. 3 groups of three guts were analysed. From these samples, the total microbial DNA was extracted using the Fast DNA SPIN Kit for Soil (MP Biomedicals, USA) with the following modifications: each sample was homogenized in the FastPrep for 40 s at speed setting of 6.5 twice, keeping it in ice between the two homogenisation steps, while the final centrifugation was carried out at 14,000×*g* for 15 min, and the final resuspension of the Binding Matrix was carried out in 50 µL of nuclease-free water.

The DNA extractions were checked with electrophoresis on a 1% agarose gel, and then quantified using a QuBit fluorometer (Invitrogen, United Kingdom).

#### DNA amplification

The V3-V4 region of the bacterial 16S rRNA gene (between 480 and 490 bp) was amplified by PCR using the universal primers 343f. (5′-TACGGRAGGCAGCAG-3′), and 802r (5′-TACNVGGGTWTCTAATCC-3′)^67^. A two-step PCR protocol was implemented in order to reduce the possibility of generating non-specific primer annealing, as detailed in Berry et al.^68^. The PCR reaction mix comprised of 20.5 µL of MegaMix (Microzone Limited, United Kingdom), 1.25 μL of each primer (10 μM), and 2 μL (1 ng/μL concentration) of DNA template. Thermal cycling conditions were as follows: Step 1: an initial denaturation at 94 °C for 5 min, followed by 25 cycles at 94 °C for 30 s, 50 °C for 30 s, 72 °C for 30 s, followed by a final extension at 72 °C for 10 min. Step 2: initial hold at 95 °C for 5 min, followed by 10 cycles of 95 °C for 30 s, 50 °C for 30 s, and 30 °C for 30 s; then, a final extension at 72 °C for 10 min. At the second step, each sample was amplified using a dedicated forward primer with a 9- base extension at the 5′ end, which acts as a tag, in order to make simultaneous analyses of all samples in a single sequencing run possible The DNA amplifications were checked with electrophoresis on a 1% agarose gel, and then quantified using a QuBit fluorometer (Invitrogen, United Kingdom). PCR products generated from the second step were multiplexed as a single pool using equivalent molecular weights (20 ng). The pool was then purified using the solid phase reversible immobilization (SPRI) method with Agencourt AMPure XP kit (REF A63880, Beckman Coulter, Milan, Italy), then sequenced by Fasteris S.A. (Geneva, Switzerland). The TruSeq DNA sample preparation kit (REF 15026486, Illumina Inc, San Diego, CA) was used for amplicon library preparation, whereas the sequencing was carried out with the MiSeq Illumina instrument (Illumina Inc., San Diego, CA) generating 300 bp paired-end reads.

### Sequence data preparation and statistical analyses

High-throughput sequencing data filtering, multiplexing and preparation for concomitant statistical analyses were carried out as previously detailed^69^. In summary, paired-reads were assembled to reconstruct the full V3-V4 amplicons using the “pandaseq” script^70^ with a maximum of 2 allowed mismatches and at least 30 bp of overlap between the read pairs. was then carried out with the Fastx-toolkit was then employed for samples demultiplexing (http://hannonlab.cshl.edu/fastx_toolkit/).

Mothur v.1.32.1^71^ was applied in order to remove sequences with large homopolymers (≥ 10), sequences that did not align within the targeted V3-V4 region, chimeric sequences^72^ and sequences not classified as bacterial after alignment against the Mothur version of the RDP training data set. Mothur and R (http://www.R-proje ct.org/) were employed to analyze the resulting high-quality sequences following the operational taxonomic unit (OTU) and the taxonomy-based approach. For the OTU approach, sequences were first aligned against the SILVA reference aligned database for bacteria^73^ using the NAST algorithm and a kmer approach^74,75^, and then clustered at the 3% distance using the average linkage algorithm. OTUs having a sum of their abundances across all samples of than 0.1% of the total were grouped into a single “rare OTUs” group. For taxonomy based analyses, sequences were classified into taxa using an amended version of the Greengenes database^76^.

Mothur and R were also employed for statistical analyses on OTU and taxonomy matrixes using hierarchical clustering with the average linkage algorithm at different taxonomic levels, Principal component analysis (PCA) for unconstrained samples grouping, Canonical correspondence analyses (CCA) to assess the significance of different treatments on the analysed diversity. Features that were significantly different between treatments were identified with Metastats^76^.

Sequence data were submitted to the National Centre for Biotechnology Information Sequence Read Archive (BioProject accession number PRJNA693145).

## Supplementary Information

Supplementary Information
