# Supplementary material for: Acute and chronic effects of Titanium dioxide (TiO2) PM1 on honey bee gut microbiota under laboratory conditions
Source: Sci Rep. 2021 Mar 15;11:5946. doi: 10.1038/s41598-021-85153-1 (PMC7960711; doi:10.1038/s41598-021-85153-1)
Supplement: Supplementary file 1 — Supplementary Information [file 41598_2021_85153_MOESM1_ESM.docx]

Acute and chronic effects of Titanium dioxide (TiO_2_) PM_1_ on honey bee gut microbiota under laboratory conditions

Papa G^1^, Di Prisco G.^2,3,4^, Spini G.^5^, Puglisi E.^5*^, Negri I. ^1^

^1^ Department of Sustainable Crop Production – DIPROVES, Università Cattolica del Sacro Cuore Via Emilia Parmense 84, 29122 Piacenza, Italy

^2^Institute for Sustainable Plant Protection, National Research Council, Piazzale Enrico Fermi 1, 80055 Portici, Naples, Italy

^3^CREA Research Centre for Agriculture and Environment, Via di Corticella 133, 40128 Bologna, Italy

^4^ Department of Agricultural Sciences – University of Napoli Federico II, Via Università 100, 80055 Portici, Italy.

^5^Department for Sustainable Food Process – DISTAS, Università Cattolica del Sacro Cuore, Via Emilia Parmense 84, 29122 Piacenza, Italy

*Corresponding author: edoardo.puglisi@unicatt.it

## Supplementary Information

**Figure S1.** Haemolymph control drop.

A) SEM image of haemolymph with haemocytes (light spots). B) EDX spectrum showing the absence of TiO2 in haemolymph.

**Figure S2.** Principal component analysis (PCA) on the total measured OTUs highlighting possible groupings of honey bee gut bacterial communities according to the treatment or the dose.
